# Public Health Policy and Experience of the 2009 H1N1 Influenza Pandemic in Pune, India

**DOI:** 10.15171/ijhpm.2017.54

**Published:** 2017-05-09

**Authors:** Vidula Purohit, Abhay Kudale, Neisha Sundaram, Saju Joseph, Christian Schaetti, Mitchell G. Weiss

**Affiliations:** ^1^The Maharashtra Association of Anthropological Sciences, Centre for Health Research and Development, Pune, India.; ^2^Savitribai Phule Pune University, Pune, India.; ^3^Department of Epidemiology and Public Health, Swiss Tropical and Public Health Institute, Basel, Switzerland.; ^4^University of Basel, Basel, Switzerland.; ^5^Saw Swee Hock School of Public Health, National University of Singapore, Singapore, Singapore.

**Keywords:** Influenza, H1N1, Pandemic Preparedness Plans, Local-Level Pandemic Response, India

## Abstract

**Background:** Prior experience and the persisting threat of influenza pandemic indicate the need for global and local preparedness and public health response capacity. The pandemic of 2009 highlighted the importance of such planning and the value of prior efforts at all levels. Our review of the public health response to this pandemic in Pune, India, considers the challenges of integrating global and national strategies in local programmes and lessons learned for influenza pandemic preparedness.

**Methods:** Global, national and local pandemic preparedness and response plans have been reviewed. In-depth interviews were undertaken with district health policy-makers and administrators who coordinated the pandemic response in Pune.

**Results:** In the absence of a comprehensive district-level pandemic preparedness plan, the response had to be improvised. Media reporting of the influenza pandemic and inaccurate information that was reported at times contributed to anxiety in the general public and to widespread fear and panic. Additional challenges included inadequate public health services and reluctance of private healthcare providers to treat people with flu-like symptoms. Policy-makers developed a response strategy that they referred to as the Pune plan, which relied on powers sanctioned by the Epidemic Act of 1897 and resources made available by the union health ministry, state health department and a government diagnostic laboratory in Pune.

**Conclusion:** The World Health Organization’s (WHO’s) global strategy for pandemic control focuses on national planning, but state-level and local experience in a large nation like India shows how national planning may be adapted and implemented. The priority of local experience and requirements does not negate the need for higher level planning. It does, however, indicate the importance of local adaptability as an essential feature of the planning process. Experience and the implicit Pune plan that emerged are relevant for pandemic preparedness and other public health emergencies.

## Background

Influenza is notable for its health and economic impact.^1,2^ It occurs in two epidemiological forms; epidemics and pandemics. Annual epidemics of seasonal influenza result in 3–5 million cases of severe illness and between 250 000 and 500 000 deaths worldwide.^3^ The economic impact includes direct costs (medicines, hospitalization), indirect costs (absenteeism and hampered productivity) and intangible costs of suffering (pain and reduced quality of life).^4^ Influenza pandemics are infrequent compared with seasonal influenza, but they are widely feared public health emergencies because they entail serious social disruption and substantial economic cost.^5^ Influenza pandemics are characterized by severe illness and high mortality, and cases typically affect not only high-risk groups but also others in the population, including young healthy adults, who are less vulnerable to seasonal influenza.^6^

Pune district in Maharashtra, India—both the urban and rural areas—has a history of seasonal and pandemic influenza outbreaks.^7^ Although data on morbidity and mortality are limited, available evidence shows that pregnant women and children under 5 years-of-age are typically among the most severely affected by regular influenza outbreaks.^8,9^ The district was the epicentre of the 2009 H1N1 influenza pandemic in India, popularly referred to as “swine flu.”^10^ A study assessing its impact documented substantial hospitalization (8.6% of cases) and mortality, with one death per 11 confirmed hospitalized cases.^11^

The severe impact of the prior influenza pandemics of 1918, 1957, and 1968 highlighted the need for preparedness and public health capacity.^8,12,13^ Other pandemic threats since the year 2000—severe acute respiratory syndrome (SARS) in 2003 and avian influenza A (H5N1) beginning in Southeast Asia in 2004, which spread globally the following year—further underscored the importance of containment strategies and public health response capacity.^12,14,15^ Although its impact was milder than anticipated, the influenza pandemic of 2009 was the first of the 21st century.^16^

Since its inception in 1948, the World Health Organization (WHO) has acknowledged responsibilities for developing and updating strategies to contain and control both pandemic and seasonal influenza through global preparedness and national programmes.^17^ Although pandemics are global, the WHO encourages member nations to develop their own national influenza programmes based on WHO’s guidance for pandemic preparedness and control of influenza that includes a planning checklist covering essential and desirable elements of pandemic preparedness.^18,19^ Nearly 100 countries made use of their own national plans in their response to the 2009 H1N1 influenza pandemic.^20,21^ Only a few countries—mostly European countries and one Southeast Asian country, Thailand—have updated their plans based on the 2009 H1N1 influenza pandemic experience.Such revision focussed mainly on assessing the impact of pharmaceutical, medical and non-medical intervention.^20,22,23^ Updated plans did not explicitly discuss questions concerning integration of global, national and local policies and their implementation,^13,19^ and the process of adapting global strategies in local programmes remains challenging.^1,14,24,25^ This question has motivated our study.

## Study Aims

This study was undertaken to examine public health experience in the 2009 H1N1 influenza pandemic in Pune. It considered relevant policy at various levels and implications for development of what policy-makers refer to as either the *Pune model* or* Pune plan* for pandemic preparedness and response. The specific aims were as follows:

1. Review authoritative pandemic influenza planning strategies at global, national and state levels.
2. Present key points in the course and response to the 2009 H1N1 pandemic in Pune.
3. Explain the provisions of the Pune plan and its relevance for preparedness for future pandemics.
4. Examine policy-makers’ reflections on the pandemic experiences and pandemic planning.

## Methods

Our analysis of the policy response to the 2009 H1N1 influenza pandemic in Pune included a documentary review and in-depth interviews of concerned policy-makers and administrators. Relevant documents indicating global, national and local level pandemic preparedness and response plans were identified and reviewed, and in-depth interviews conducted with administrators and public health officials who coordinated the pandemic response in Pune.

### Study Setting and Design

The tropical monsoon climate of Pune district is associated with regular outbreaks of seasonal influenza.^26^ The district was more seriously affected, however, by the 2009 H1N1 influenza pandemic.^10,11^ Moreover, Pune is not just an influenza problem site; it is also the site of key resources such as a national laboratory where virological testing was done during the 2009 H1N1 influenza pandemic. Furthermore, a major vaccine production facility, the Serum Institute of India (SII) is located in Pune. The SII was asked to develop a live attenuated influenza vaccine (LAIV) in the framework of the WHO’s effort to strengthen influenza vaccine production in developing countries. The experience of the 2009 H1N1 influenza pandemic in Pune was a focus of attention for the entire country, and it served as a test case for India’s preparedness and response in real time.

### Review of Planning Documents

Our review of planning documents identified essential features of influenza public health policy at local, national and global levels of pandemic planning. We focussed on authoritative primary source planning documents at each level rather than a systematic review of the secondary literature. Consequently, key documents reviewed included a WHO guidance document for pandemic influenza preparedness and response,^27^ the Government of India’s action plan for H1N1 influenza,^28^ and a Maharashtra state contingency planning document (see Supplementary file). These documents were downloaded from the respective websites of the WHO; the Ministry of Health and Family Welfare, Government of India (MoHFW, GoI); and the Maharashtra Public Health Department.

### Key Informant Interviews

Health system contacts, policy documents and news coverage during the period from June 2009 to August 2010 enabled us to identify eight public health officials and administrators for key informant interviews. An open-ended interview agenda was formulated to address our study objectives, inquiring about the response to the pandemic over its course with regard to planning and implementation. We then asked the respondents to reflect on the process and its effectiveness, and to consider implications for future pandemic planning. We also asked them about details of development and implementation of the strategic response plan, challenges of essential tasks, experience of the planning process, lessons learned and consideration of implications regarding preparedness planning for future pandemics. The interview agendas are provided in Appendix 1 and Appendix 2. Two researchers were trained to conduct these interviews in *Marathi (local language)*, and an informed consent form was prepared to obtain respondents’ permission for the interviews and audio recording.

### Data Management and Analysis

Our analysis proceeded in three steps. We first extracted key elements of the pandemic preparedness and response plans from the policy documents to characterize the strategy for containment and control. Next we summarized the course of the Pune pandemic experience and milestones that defined periods for which particular policy priorities were relevant, from April 2009 to August 2010. Finally, we reviewed key elements of the policy response and implementation based on data from in-depth interviews with regard to experience and reflections of the policy-makers.

## Results

The results are presented into two parts, first with a review of global, national and state plans considering implications for the district-level planning. Second, we focus on experience in Pune—reviewing the course of the pandemic, the public health response, features of the Pune plan that emerged and reflections of policy-maker key informants.

### Global, National and State Plans for Influenza Pandemic Response

#### World Health Organization’s Global Influenza Programme

The WHO’s guide for global and national preparedness and response^27^ considered the periods before, during and after local outbreaks of the global pandemic.^18^ It was intended to serve as a guide, but not replacement, for national plans. The document explained the role of the WHO, and recommendations were presented with reference to six phases of a pandemic. These include phases 1–3 of predominantly animal infections and a few human infections. During these earlier phases, countries are expected to exercise and review national pandemic preparedness and response plans, develop a robust surveillance system, promote preventive public health measures, prepare for health system scale-up and complete a communications plan to communicate risks and provide regular updates about the course of the pandemic. During phase 4, which is characterized by sustained human-to-human infection, countries should implement containment measures by activating a contingency plan, increase surveillance, and promote recommended public health measures to prevent or reduce risk of infection. Phases 5 and 6 involve widespread human infection, during which resources are required to implement coherent and comprehensive containment and mitigation strategies.

#### National and State-Level Preparedness Plans With Reference to the Global Framework for Pandemic Phases 5 and 6

Table 1 presents key activities of phases 5 and 6 that are documented in global, national and state-level plans for influenza control. Based on the framework indicated by the WHO, it indicates five critical considerations for preparedness and response across global, national and state levels.

**Table 1:** Preparedness and Response Components for Phases 5 and 6 at Global, National and State Levels

**Preparedness and Response**	**Global**	**National (India)**	**State (Maharashtra)**
Leadership and coordination of multi-sectoral resources	Provide leadership and coordination to multi-sectoral resources	Guidelines for sectoral co-ordination, institutional framework at national, state and district levels indicating roles and responsibilities	Description of institutional framework for Maharashtra state
Monitoring and assessment of evolving pandemic: impact and mitigating measures	Active monitoring and assessment of evolving pandemic, its impact and mitigating measures	Mechanism for strengthening surveillance, laboratory support and airport screening during phases 5 and 6	Description of operational aspects of active and passive surveillance and identifying suspected cases
Behavioural, social and pharmaceutical interventions	Implement individual, societal and pharmaceutical measures	Mechanisms for case investigation, infection control practices, pharmaceutical and non-pharmaceutical interventions	Guidelines for implementing public health measures and details regarding planning logistics, and supply of equipment and drugs
Implementation of health system contingency plans	Implement contingency plans for health systems at all levels	Mechanisms for training healthcare staff and mobilizing the healthcare system to cope with influx of patients	Guidelines for clinical management and training paramedical and medical staff
Update general public and stakeholders	Ongoing updates to general public and all stakeholders	Communicating situation-specific information to general public to minimize fear and promote appropriate health-seeking behaviour	Guidelines for establishing a control room and appointing a spokesperson

According to the WHO guide, advanced pandemic phases 5 and 6 are declared when sustained community-level outbreaks caused by the same influenza virus have been identified in two or more countries in one WHO region (phase 5), and in another country in another WHO region (phase 6).^27^ In phases 5 and 6, countries should provide leadership and multi-sectoral coordination, implement mitigation measures specified in the national plan, and provide updates to the public and other stakeholders on the course of the pandemic and measures implemented to mitigate its threat. During the pandemic — but after the peak — experience should be evaluated, and plans for preparedness and response should be updated based on lessons learned.

#### India’s National Plan

A national Action Plan for Pandemic Preparedness and Response^28^ was prepared in the MoHFW, GoI, and published in June 2009 by the Directorate General of Health Services (DGHS). This document presented India’s national policy plan for influenza pandemic management, and it focused on the priorities of phases 5 and 6 of the WHO guide. Development of the Indian plan also relied on experience adapted from the *Contingency Plan for Management of Human Cases of Avian Influenza*, published by the DGHS in 2005.^29^

The plan for managing pandemic influenza included strategies for early detection, appropriate case management and public health measures based on guidelines, protocols and standard operating procedures (SoPs). It presented contingent strategies to address two scenarios, according to whether or not the pandemic had already reached India. These strategies were formulated with reference to the five-fold framework of the WHO guide.^27^

The national action plan intended to provide a practical framework to guide states that were implementing strategies for their districts. Some points in the WHO guide, however, were not addressed. There was no provision for testing and revising the national plan, and recommendations of the WHO checklist for legal and ethical issues were not considered in the national plan.

#### Maharashtra’s State Plan

The state of Maharashtra also published its *Contingency Plan for Management of Influenza A H1N1* in June 2009 by the Directorate of Health Services (see Supplementary file).It described the state-level institutional framework and how the state and districts should coordinate management of the influenza pandemic.

Although the state’s plan was expected to provide operational guidelines and training for capacity building of the districts, the state plan only described needed action in response to the pandemic. Operational guidelines indicating responsibilities and how recommended action might be implemented were not detailed. Furthermore, mechanisms for review and revision of plans were not covered in the state-level plan. The document did mention that the plan would be sent to responsible offices of the Central Government, such as the MoHFW, GoI, and the National Disaster Management Authorities. Nevertheless, details of centre and state interactions to enable solicitation and incorporation of comments and advice from the central government were lacking.

#### Framework From Global, National and State Plans for Analysing a District-Level Response

**Figure 1:**
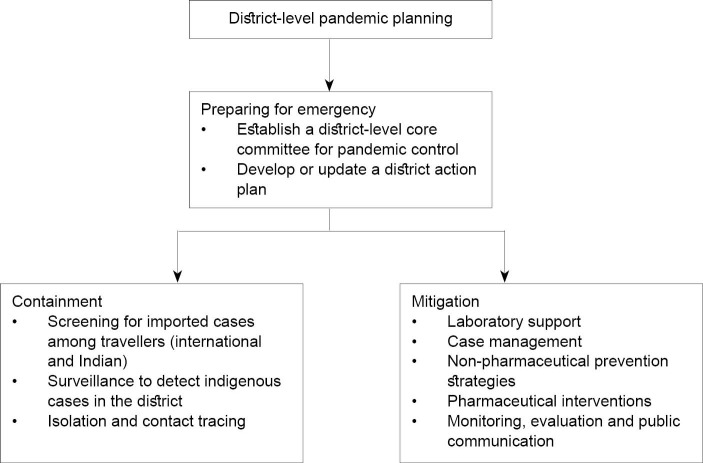
Framework for Analysing District-Level Public Health Response to Influenza Pandemic.

The planning documents indicate particular priorities at the various stages of pandemic experience, acknowledging that each poses distinct challenges. Initially, the threat of pandemic in the early stages of the outbreak requires strengthening capacity of the health system to respond with containment and mitigation strategies well before the full outbreak ensues. Preparing for a public health emergency should begin much earlier. Containment and mitigation strategies become ever more compelling priorities as the outbreak becomes more severe. An overview of these considerations for district-level planning, which also acknowledges the framework of the three levels of policy documents summarized above, is outlined in Figure 1. Such considerations are relevant for analysis of the influenza pandemic experience, the Pune plan and reflections of policy-makers.

### Elaboration of the Course and Response to the Pandemic in Pune

#### Key Informants

We initially contacted eight public health officials and administrators identified from policy documents and news coverage in September 2013 through telephone calls and personal visits. Five were available and agreeable to participate in key informant interviews, which were completed by June 2014. These key informants included the chief administrative officer (CAO) of Pune district, the former assistant administrative officer of Pune city, the city medical officer, the former in-charge official of the government-run infectious diseases hospital and a senior officer of the government diagnostic laboratory, Pune.

A study information sheet was given to all respondents before their interviews. Verbal consent included an agreement that respondents would not be identified by name without explicit permission, although roles and generalized designations are specified to indicate the rationale for selection and perspectives represented by respondents. The average duration of these interviews was one hour. All but one of the interviews were audio recorded, and for the respondent who agreed to the interview but declined audio recording, hand-written interview notes were prepared. Seven interviews were conducted at the hospital or office workplace of the respondents, and the interview with a former assistant administrative officer of Pune city was conducted at his residence.

#### Course of the Pandemic

**Figure 2:**
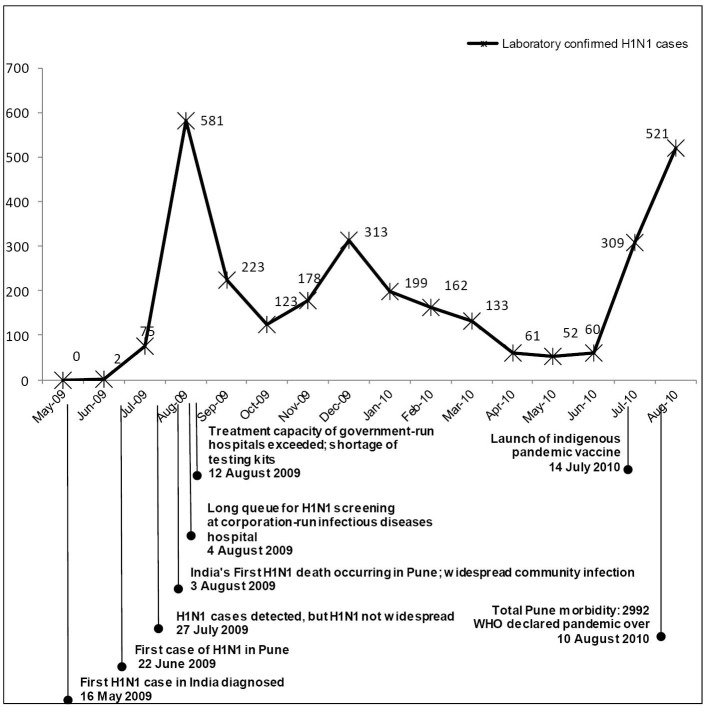
Progression and Confirmed Morbidity of the 2009 H1N1 Influenza Pandemic in Pune.
Source of morbidity data: Health Department, Pune Municipal Corporation.

The pandemic events span the period from May 2009 when the first cases were diagnosed in India to August 2010 when WHO declared the pandemic was over. The events sketched in Figure 2 and elaborated in the text below were gleaned from the interviews and media reports.

On May 16, 2009 the first case of pandemic influenza in India was detected in Hyderabad. A month later, a software engineer returning to Pune from the United States was admitted to the infectious diseases hospital in Pune. On June 22, 2009, he became the first imported case of H1N1 influenza in Pune.^30^ Sporadic cases of H1N1 infection were diagnosed through the end of July 2009, but transmission was not widespread. An outbreak in a school situated in a highly populated Western suburb of Pune was widely publicised by the media during this period.

The first influenza pandemic death in India occurred in a private hospital in Pune on August 3, 2009. Widespread community infection followed soon after that, accompanied by panic fuelled by a lack of authoritative information about the risk of infection. The family of the deceased accused the hospital of negligence and filed a case in the consumer court. Newspapers gave extensive coverage to the case. This made private doctors reluctant to treat patients with flu-like symptoms, and after 4 August, long queues of symptomatic patients were being seen at three government hospitals in Pune.^31^ Within a week, facing shortages of testing kits, these hospitals had no further capacity for testing and admitting patients.

Medical stores in Pune also faced shortages of supplies, such as sanitizers, eucalyptus oil and vitamin C tablets, which were popularly thought to afford protection from H1N1 influenza.^32^ At this point public demand for vaccination was high, but vaccines were unavailable. Confusion and chaos resulted from a lack of public understanding about the diagnosis, treatment and prevention, and it persisted through August 2009. A year later, the WHO declared the pandemic to be over.

#### Public Health Response

Key features of the public health and administrative response over the course of the outbreak are outlined in Table 2 and elaborated in the text that follows.

**Table 2:** Public Health Response to Influenza Pandemic Experience in Pune

**Date/Period**	**Public Health Response**
May 2009	Pune's Public Health Department began preparing pandemic response
May 16, 2009–Aug 2, 2009	Airport screening, contact tracing and other strategies implemented
Aug 4, 2009	Maharashtra State Government invoked the Epidemic Act of 1897 in Pune district to control the pandemic
Aug 5, 2009	PSFMC constituted
Aug 6, 2009	PSFMC developed an action plan to contain influenza pandemic
Aug 6, 2009–Aug 18, 2009	Screening centres established throughout the city
Aug 6, 2009	SoPs prepared in government-run tertiary care hospital for managing influenza pandemic patients
Aug 6, 2009–Aug 13, 2009	Private hospitals with ICU facilities identified and engaged for seriously ill influenza pandemic patients
Aug 6, 2009–Aug 13, 2009	Standard reporting system developed for pandemic cases, deaths and treated patients
Aug 6, 2009–Aug 8, 2009	Media briefing policy implemented for authoritative information on prevention, help seeking and treatment over the course of pandemic
Aug 11, 2009–Aug 25, 2009	Educational institutions and entertainment facilities closed for 2 weeks
Nov 2009	District-level action plan prepared for wave II of influenza pandemic
July 14, 2010	Launch of locally developed and produced LAIV by SII, located in Pune
June 22, 2009–Aug 10, 2010	Real-time PCR technology at government diagnostic laboratory made available for rapid and accurate diagnosis

Abbreviations: PSFMC, Pune Swine Flu Management Committee; LAIV, live attenuated influenza vaccine; SII, Serum Institute of India; PCR, polymerase chain reaction; ICU, intensive care unit; SoPs, standard operating procedures. Data sources: Primarily key informant interviews and supplemented by media reports.

After the first H1N1 case in India was announced, Pune’s public health department recognized that it was needed to prepare for an emergency and immediately began planning a pandemic response. The local health department assumed major responsibility for this containment strategy before widespread outbreak. It focused primarily on stockpiling antivirals and personal protective equipment (PPE, viz, masks and gloves). The public health officials we interviewed in Pune explained that it was clear when the first influenza pandemic case was diagnosed in Hyderabad, that Pune would surely face a similar threat. Climate and international contacts through the IT industry were notable, which contributed to vulnerability. Pune’s public health department was in close contact with the central government, particularly with the national institute in Delhi responsible for planning and implementing the public health response to communicable disease emergencies.

At this early stage of the outbreak, Pune district did not have its own pandemic response plan, and so it relied on guidance of the central and state governments. Although the importance of disseminating timely and accurate information for the public was recognized, efforts were inadequate to forestall growing concerns and mounting public anxiety. After WHO classified the pandemic as phase 5 in the first week of May 2009, the Government of India began screening international passengers at all of the country’s international airports. This early measure aimed to detect infected persons who might spark transmission. By June 5, 2009, of the 1229 passengers who arrived on international flights in Pune, at least 11 suspected cases with symptoms of pandemic influenza had voluntarily reported to the local government’s infectious diseases hospital in Pune and were isolated for a day. None of them, however, tested positive for the virus.

After H1N1 cases were diagnosed in various parts of the country in June, nine surveillance centres, including one in Pune, were set up by the national diagnostic laboratory. They were established to detect indigenous cases, especially among people in contact with confirmed cases. Although our respondents could not specify precise numbers and such data are unavailable, this approach was a priority in the early efforts to contain the outbreak.

With support from the state, the Pune public health department set up a 20-bed isolation ward at the local government infectious diseases hospital in the month of May 2009. This was the first and only designated isolation ward for H1N1 cases. According to the official who had been in charge, the ward was small but it conformed to government guidelines, and it was in other ways an ideal setup for an isolation ward with good ventilation and protected access. According to policy, all cases of confirmed pandemic influenza were quarantined at the local government’s infectious diseases hospital for one week and treated with the antiviral drug Tamiflu (Osletamivir). Close contacts of these cases were traced and given Tamiflu as chemoprophylaxis. Our informant advised that the procedures for isolation and contact tracing were meticulously implemented.

On July 27, 2009 the CAO of Pune district, who was responsible for coordinating the public health response during the pandemic, held the first press conference to assure the public that the situation was under control. Up to the last week of July 2009, there had been 55 pandemic influenza cases. His message asserted that management of these cases and follow up identifying their close contacts was proceeding according to the prescribed protocol of the central government.

#### Public Anxiety Required Further Action

After the first Indian pandemic death on August 3, 2009 in Pune, the level of concern rapidly escalated. In the absence of adequate information about what was happening and what to do, long queues of people with flu-like symptoms presented at the city’s infectious diseases hospital. The officer in charge estimated that after 3 August, “There were nearly six to seven thousand people every day in the regular Out-Patient Department (OPD).” Print and television media publicized these developments. On 7 August, The *Indian Express* newspaper wrote that “The infectious diseases Hospital was flooded with citizens for the past two days owing to a sudden rush after many general practitioners sent the patients to the hospital.”^33^ Press coverage criticised the health system’s lack of preparedness and capacity to respond to the pandemic.

Media attention and public concern demanded urgent action from the government. In the absence of an adequate pandemic response plan for the district, preparing one became a top priority. Two days after the first H1N1 influenza fatality, the Maharashtra government invoked the Epidemic Act of 1897 in Pune district. This act empowers centre, state or district administrators to issue regulations and impose measures to limit the spread of infection in the country, state or district. After invoking the Epidemic Act, failure to comply with sanctioned policy became a punishable offense under Indian Penal Code.^34^

The CAO took charge of coordinating the pandemic response for Pune district. Although the vintage law from more than a century ago helped to raise public awareness and provided substantial power, its limitations were also notable. He explained:

“*It helped to make people aware … It was a good tool enabling the administration to engage private or government hospitals for any preventive measures or treatment services. But the act is old and lacks needed guidelines for responding to the epidemic. To control the situation in Pune we had to devise mechanisms for two broad aims: for prevention and containment of infection and for treatment of the infected people.”*

Whatever its limitations, invoking the Epidemic Act of 1897 authorized the district administration of urban and rural Pune to work together. The district administration and local health department convened and conducted joint meetings of a collective body known as the Pune Swine Flu Management Committee (PSFMC).

As efforts to formulate a Pune plan were developing, the media, elected officials of city government (corporators) and heads of medical education institutions advocated for implementation of the Mexico model, which involved cordoning off the whole city.^35^ After reviewing and scrutinising this approach, the PSFMC concluded that such drastic measures—restricting movement of people in public places and travel into and out of the city—were impractical and could not be implemented in Pune. In the absence of alternative models or guidelines for managing this health crisis, the management committee developed a strategy for Pune district based on key elements of existing frameworks. Responding to the public outcry and over a period of 10 days from 3 August, centres were established for screening, and to prescribe and provide Tamiflu tablets for symptomatic patients without waiting for results of diagnostic swabs. Schools and cinema halls were closed, and development of a coordinated pandemic management strategy for Pune district proceeded at an accelerated pace.

#### The Pune Plan

The coordinated strategy that emerged from the ensuing process was designated by our policy-maker respondents as the *Pune plan* of pandemic response. It began by establishing 51 testing centres across the city to reduce the burden on staff of infectious diseases hospitals run by the Pune Municipal Corporation. The Pune plan elaborated elements of the mitigation strategy noted in Figure 1, consistent with the global, national and state plans. This framework included laboratory support, clinical case management, non-pharmaceutical prevention strategies, pharmaceutical interventions, outbreak monitoring, and public communication.

*1. Laboratory support:* The laboratory facility of the government diagnostic laboratory in Pune was a unique resource contributing to the city’s pandemic response capacity. From May 2009, a nasal or throat swab from each suspected patient with flu-like symptoms was tested for H1N1 influenza virus. Real-time polymerase chain reaction (PCR) technology was used for accurate testing, and in most cases lab results were available within 12 hours through a specially developed SMS-based system.^28^ This diagnostic laboratory facility not only enabled timely clinical diagnosis, it also provided a capacity for monitoring and research to guide policy. Data from the diagnostic laboratory supported discontinuation of contact tracing as infections became widespread. They also informed criteria to determine who should receive treatment with Tamiflu.

*2. Case management:* Fear of punishment for misdiagnosis and mismanagement of H1N1 cases made private medical providers reluctant to see suspected cases, and so they referred many more than they otherwise would to the government infectious diseases hospital. As the number of hospitalized cases increased, two more government hospitals were designated to manage pandemic influenza, one managed by the local Pune Municipal Corporation and the other a state-run tertiary care hospital in Pune. The committee decided that patients without complications would be managed at this local government hospital, and those who needed a higher level of care would be managed at the state-run tertiary care hospital.

An additional pandemic care centre with 15 beds was established at a state government-run tertiary care hospital in the city. These beds were in a unit isolated from other wards, well-ventilated and well-lit. It also had a separate area for entry and exit, a central oxygen supply and sufficient ventilators. The number of medical and paramedical staff in Pune was insufficient for the round-the-clock influx of influenza pandemic patients, and the Maharashtra State government deployed 200-300 medical and paramedical staff from other districts. The additional clinical staff supported both government and private hospitals. PPE (masks and gloves) and Tamiflu tablets were supplied by the Maharashtra State.

Three tertiary care government hospitals could not meet increasing needs of urban Pune and surrounding areas for testing and hospitalization. The PSFMC also identified thirteen private hospitals with intensive care units (ICUs), and following the protocol of the national plan, isolation wards were set-up in these hospitals.

Morbidity and mortality data were consolidated from all centres for monitoring and evaluation. Standard formats were developed for H1N1-positive case reporting, hospital admissions, patients who required ventilators, the distribution of Tamiflu tablets and H1N1 deaths. The PSFMC required all hospitals seeing H1N1-positive cases to report these details on a daily basis using, either on a prescribed paper form or using an electronic format made available to them.

*3. Non-pharmaceutical interventions:* As the outbreak progressed, the PSFMC developed a range of preventive public health measures to limit transmission. Some were based on minimising contact in institutions and communities (eg, closing schools, cinema halls, markets and malls). The committee conducted meetings with the chief operating officers or other leaders of these institutions and ordered them to close their facility for 15 days; other measures focussed on personal risk behaviours (eg, handwashing and cough etiquette). Relevant information was disseminated through print and electronic media. Public cooperation was important to motivate support for inconvenient interventions, such as closing public institutions. Media were enlisted to explain the rationale for limiting and participating in public gatherings, as a curfew-like situation prevailed for almost two weeks in Pune city until the end of August 2009.

*4. Pharmaceutical interventions: antivirals and vaccine:* Antivirals and vaccines are important resources for control of influenza. After the country’s first death and the proliferation of cases that followed, Pune received a bulk supply of the antiviral Tamiflu on May 10, 2009 from the one million tablets allotted to the state government. An adequate supply of Tamiflu was available in Pune throughout the course of the pandemic.

The Government of India made an imported inactivated monovalent vaccine available for healthcare staff in March 2010. The indigenous LAIV became available in India later, in July 2010. Although the more costly imported vaccine was provided without charge, respondents explained that many medical officers—including doctors, and other healthcare staff—did not take the vaccine. Misconceptions exaggerating risk of Guillain-Barré syndrome and the erroneous idea that prior exposure to seasonal influenza had already made healthcare providers immune were reasons offered for vaccine refusal and low uptake among healthcare staff. A city medical officer explained,

“*Injectable vaccine was available. It was for healthcare staff. But nobody was ready to take the vaccine. We had received 2000 doses of injectable swine flu vaccine from Delhi. Even though we made it compulsory only 100 persons took the injectable vaccine.”*

Development of the indigenous vaccine (LAIV) resulted from an initiative of the WHO and the presence of the SII, a major vaccine manufacturer in Pune. The WHO provided the LAIV technology to the SII in July 2009, which enabled development and production of ‘Nasovac.’^36^ This was the first indigenously produced vaccine in India against H1N1 influenza, and it became available for use in July 2010.^37^ Although vaccine uptake was high in 2010 and 2011, from 2011 in the aftermath of the pandemic, uptake dropped. The pandemic had been declared officially to be over, and no guidelines or priority existed to recommend this or other vaccines for prevention of seasonal influenza. Even though infection continued and still persists, fear of influenza pandemic in the general population had abated. A health officer of the Pune Municipal Corporation explained,

“*People were very scared in 2009-2010, but slowly the number of (H1N1) cases decreased. By 2012 there were very few cases, and people had almost forgotten about swine flu. This swine flu has now become endemic.”*

*5. Outbreak monitoring, evaluation and public communication:* Accurate and timely epidemic intelligence and public information is critical for managing any infectious disease outbreak, but no such information was available in Pune until the end of August 2009. The former CAO explained the complexity of the task of compiling, analysing, using and reporting relevant data:

“*There were three government hospitals, many private hospitals, many beds [in addition to those designated for H1N1 influenza patients] and many isolation wards. But within a few weeks we devised the mechanism to collate the data from all private and government hospitals.*”

Elaborating further, he explained that in the early days of the pandemic, the single press conference held at the end of July had not been enough to explain the issues and assure the public that the situation would be controlled. After invoking the Epidemic Act, a strategy for media briefings was developed. The PSFMC gave daily updates to media representatives covering morbidity, mortality and prevention measures. A group of spokespersons was organized for daily information outreach to the public through the media. These efforts to explain the public health response were intended to build public trust and credibility for the district administration.

#### Key Informants’ Reflection and Assessment of the Pune Plan

Our informants acknowledged the need for pandemic preparedness planning and problems resulting from its initial absence. They also acknowledged a tendency in Indian public health services to ignore planning needs until a crisis demanded a response. The former in-charge of the government-run infectious diseases hospital elaborated the point as follows:

“*We Indians are better at dealing with a crisis situation, meaning we can tackle the crisis very well but we don’t have a long term planning. Though this experience has increased awareness about influenza illness, it did not initiate any fundamental change at Government level with regard to influenza pandemic preparedness. Still we don’t have public health machinery to document the influenza burden. In our country from state to district level, health is never considered as an important subject. As a result, we don’t do any planning, and ultimately it leads to delay in taking decisions. It’s a red tape system, so nobody takes initiative unless told to.*”

Some positive effects on planning, however, also resulted from the pandemic experience. The struggle to set up an appropriate response system and the lessons learned during the first year of the pandemic prompted PSFMC to prepare an action plan for an anticipated second wave. The action plan for a second wave was based on prospective estimates of nearly 80 000 cases that may require hospitalization. This action plan is based on the state’s contingency plan, and it focussed on health services planning for a large number of seriously ill patients. SoPs for managing H1N1 patients were also prepared by the state government tertiary care hospital in Pune.^38^ Both were by-products of the Pune pandemic response.

## Discussion

Our review indicates the apparent difficulties and challenges of preparedness planning^15^; and this analysis brings several practical issues into focus. Our review of the background, setting and development of the Pune plan and its broader implications for pandemic planning highlights the relevance of integrating global, national and state-level policy to guide the response locally at the district level. This point is consistent with the finding of a review committee commissioned by WHO to examine the International Health Regulations (IHR 2005) and experience gained from the global pandemic of 2009, which also highlights the importance of integrating global, national and local planning.^39^

Our review explains how guidance for global, Indian national and Maharashtra state public health responses to the pandemic were rooted in these various documents published in 2009. Each level addressed similar or complementary issues, but their scope was distinctive. The WHO’s plan had been formulated to guide and support national planning. India’s national planning document aimed to support state planning, and the state plans aimed to facilitate district planning. In India, as the threat of an imminent pandemic became clear in 2009, it evoked a mix of concern about limited public health capacity and anticipation of an opportunity to rise to the challenge of preparing an effective pandemic response.^40^

Both the higher-level framework and the experience in Pune shaped development of the Pune plan. The ‘Pune plan’ that emerged in the district was a required expedient for immediate implementation. Borne of urgency imposed by the serious burden of illness affecting the city, opportunities for planning were also enabled by a unique set of public health resources. These resources include a major centre for virology with capacity for rapid real-time PCR diagnosis, presence of a major vaccine producer in Pune and a public health infrastructure that recognized a need to identify and address key tasks of pandemic preparedness. Epidemiological expertise enabled officials to monitor the course of the outbreak, a key task for control.^41^ Research studies conducted at the national diagnostic laboratory in Pune informed pandemic policy decisions and appropriate management of resources, such as the decision to start presumptive treatment and discontinue contact tracing. Several features of the Pune plan are particularly noteworthy, namely, the role of vaccines and the organization of health services.

### Vaccines

Notwithstanding the extraordinary resources for developing the LAIV in Pune with support of WHO and the resources of the SII, several issues limited the effectiveness of vaccines during the pandemic. At the outset when they were needed most, vaccines were unavailable. By the time the LAIV was developed and available, hesitancy and low uptake among health professionals and the general population limited effectiveness. Among healthcare providers, fear of serious side effects, such as Guillain-Barré syndrome or death, were the main reasons of low uptake. For the general population, however, limited coverage was more a matter of limited access, cost of the inactivated vaccine, low perceived personal risk after July 2010 when the LAIV became available, inadequate information and a perceived lack of a government mandate endorsing influenza vaccine.^42^

As a result of these constraints, despite access to the vaccine at no cost for health professionals or at low cost for the LAIV vaccine in the general population, neither was enough to ensure appropriate use. Social and cultural determinants of vaccine acceptance, both facilitators and barriers, also played a role. It is notable that coverage problems typically attributed to vaccine hesitancy of communities in Western Europe and North America were less of an issue than hesitancy among health professionals to use and prescribe the available vaccine.^42^ Implications of vaccine hesitancy among professionals extend well beyond pandemic disease control to other settings, especially with regard to prescribing practices for high-risk groups, such as pregnant women, for whom seasonal influenza vaccination has become a priority for influenza control.^3^

### Health services

The failure to ensure adequate health services capacity earlier was a result of waiting for an urgency-driven planning process of ad hoc measures. Limited local capacity was a serious problem for managing the initial surge of pandemic cases.^8,43^ Earlier recognition of needs and consideration of strategies to meet surge demands would have been useful. Phadke suggests that failure of public and private sectors to interact was a serious problem, and overly restrictive criteria for diagnostic testing of a swab (ie, history of foreign travel or close contact with a known H1N1 case) resulted in missing many cases at the beginning of the outbreak.^44^ In India there is a huge and unregulated private health sector, and private doctors are often the first point of care for many people,^45^ including people with influenza during the 2009 pandemic in Pune.^46^

A plan to involve private hospitals would have boosted capacity earlier. When the need to rethink a strategy was recognized, it took only two weeks for the local government to engage 13 private hospitals with intensive care facilities conforming to the WHO’s recommendations. The WHO influenza guidance document advises inclusion of both public and private leadership in planning for influenza preparedness and response.^27^ Lack of credible information about the influenza pandemic^40,44^ was the key factor creating panic in the public and uncertainties among the private medical providers, who also had concerns about liability arising from media coverage of the first death in a private Pune hospital. Delayed treatment contributed to mortality.

### Legal and Ethical Framework of Pandemic Response

With regard to the legal framework for pandemic response, our review shows that as planning became a priority, existing legislation enabled action in response to public health emergencies. But that legislation also required updating.^47^ The Indian Epidemic Act of 1897 was dated, and it has been criticized as archaic and blunt,^48^ but as one of our policy-maker respondents explained, for public health officials who needed to take extraordinary measures in response to the emergency, it proved to be useful. Ethical dimensions of public health action to protect human rights, which were considered in WHO’s global guide, were not discussed in the Indian national plan, although a public health authority in an article reflecting on avian flu outbreaks of 2005 suggested that it would be unethical not to employ public health tactics to prevent hardships, and that critical ethical concerns arose primarily from inadequate diagnostic capacity and services.^49^

### Priority of Planning

Our respondents acknowledged a reluctance to plan unless pressed by emergency. In the plan that emerged, consideration of assessment, feedback and revision was lacking, although it had been recommended in the higher level planning guides. As Burkle advises, pandemics begin and end at the local level, and local assessment is critical but too often neglected.^1,50^ Statutory or regulatory planning requirements at district and state levels that mandate public health action may help to mitigate pandemics and serious outbreaks in the future. An approach to planning that involves a broad group of stakeholders should be considered, including administrators, public health officers, government and private hospital clinicians, private practitioners, vaccine manufacturers, laboratory scientists, media persons and community representatives.

The influenza H1N1 virus has now become a seasonal strain, and it continues to cause deaths in all the age groups in India. Planning diagnostic and treatment healthcare services remains important, inasmuch as reports of high morbidity and mortality persist. Health education, vaccine promotion, identification of priority groups for vaccination and guidelines for managing outbreaks are required for effective control, not only of pandemic but also seasonal influenza. More serious attention to surveillance and planning and a wider role of vaccine prevention for seasonal or endemic influenza has been advocated by some authorities,^51^ primarily for selected high-risk groups, especially pregnant women. Local strategies for control in the general population, whether or not they include vaccination, are also needed,^52^ but as serious illness associated with pandemic disease has become endemic, there is a risk of increasing needs in a context of diminished priority.

## Limitations

Our documentary review focussed on relevant global, national and state-level planning documents available in public domain, but it was not a comprehensive review of pandemic preparedness planning in other regions. Interviews of the five key informants were conducted three years after the pandemic; hence there is a possibility of recall bias. The three authoritative respondents who were unavailable for the interviews may also have provided additional insights. The study was undertaken in Pune for reasons explained in the text. The epidemiology and special resources that recommended it as an influenza study site must nevertheless be acknowledged as a limitation with regard to generalizability of locally held planning priorities and competencies.

## Conclusion

The planning process for controlling pandemic influenza and persisting serious endemic influenza requires consideration of global, national and local responses. In India these responses are mediated through state- and district-level planning. Although experience varies at each of these levels, a planning process capable of acknowledging and addressing such variation is required. Our study of the experience and response to the influenza pandemic of 2009 recognized the initial panic before a planned public health response was developed and implemented as an ad hoc expedient. Measures included justifiable closure of schools, shopping centres, entertainment centres and cancellation of planned public gatherings. Such restrictions were enabled by the Epidemic Act of 1897.

The Pune plan for preparedness and response to the pandemic was a product of a management committee of public health officials. The plan addressed needs for diagnostic laboratory support, case management, non-pharmaceutical preventive measures, pharmaceutical interventions, and regular monitoring and feedback through public communications. It is broadly relevant in Pune and for consideration in India and other settings as a framework for pandemic preparedness. Our study shows the importance of district-level planning guided by higher level policy. It also indicates the challenges of implementing vaccine policy and organizing health system resources.

## Acknowledgements

We are grateful to the study participants for sharing their views and experiences. We appreciate the support of the Health Department of Pune Municipal Corporation in our study. The study was funded through the grant from the WHO’s Initiative for Vaccine Research (IVR), agreement number (U50CL000748). The authors would like to acknowledge the contributions of the US Centers for Disease Control and Prevention (CDC) which provided financial support to the WHO’s IVR.

## Ethical issues

This study was approved by the WHO Research Ethics Review Committee, Geneva, Switzerland; the MAAS Institutional Ethics Committee, Pune, India, and the Ethics Commission of Basel, Switzerland. The objectives of the study were explained to the participants and informed consent was obtained.

## Competing interests

The authors declare that they have no competing interests. The study was supported by the World Health Organization (WHO), Geneva, Switzerland. The funders had no role in study design, data collection and analysis, preparation, review or approval of the manuscript.

## Authors’ contributions

All authors participated in the study design and coordination of the study. VP, AK, and SJ undertook data collection and analysis, and VP drafted the manuscript. AK, NS, CS, and MGW contributed to revision and completion of the submitted version. All authors have reviewed the manuscript for intellectual content and have read and approved the manuscript.

## Authors’ affiliations

^1^The Maharashtra Association of Anthropological Sciences, Centre for Health Research and Development, Pune, India. ^2^Savitribai Phule Pune University, Pune, India. ^3^Department of Epidemiology and Public Health, Swiss Tropical and Public Health Institute, Basel, Switzerland. ^4^University of Basel, Basel, Switzerland. ^5^Saw Swee Hock School of Public Health, National University of Singapore, Singapore, Singapore.

## Supplementary file

Supplementary file A supplementary file has been added for the “Contingency Plan for Management of Influenza A (H1N1),” published by the Directorate of Health Services, Government of Maharashtra. This document was accessed and downloaded for analysis on July 14, 2014 from the link cited in the document: http://maha-arogya.gov.in. This link, however, is no longer active, and we provide the document as a supplementary file.

## Key messages

**Implications for policy makers**

- Evidence generated through the experience of the 2009 H1N1 influenza pandemic should be acknowledged. Analysis of the response and consideration of findings should strengthen planning for preparedness.
- Findings from our review consider strategies for ongoing activities, including continued public health surveillance of influenza, guidelines for criteria-based diagnosis and the need to ensure effective communication among all concerned stakeholders.
- Specific efforts are required to formulate national policies for influenza vaccination and to promote awareness of the status of primary healthcare providers as a high-risk group that should be vaccinated, and the priority for them to vaccinate their patients.
- Colonial legislation played a major role in developing a pandemic response in Pune, but it is important to update such legislation to acknowledge experience and support pandemic management strategies.

**Implications for public**

The threat of recurrence of an influenza pandemic indicates a need not only for vigilance and preparedness of policy-makers but also the priority of community awareness. A strategy to explain risks and recommendations to the public is a fundamental interest and responsibility of the health system for managing future outbreaks. Our analysis of experience of the 2009 pandemic in Pune and implications incorporated in the Pune plan are relevant to engage the public in pandemic response planning.

## Appendix 1

Appendix 1. In-depth Interview Guide for Local Level Health Officials

1. Qualification: Training and specialization, position(s) held
2. Description about course and development of 2009 H1N1 influenza Pandemic situation
3. How was the response strategy planned at district?
4. Your role in pandemic management?
5. Your experiences: (Individual) clinical management protocol and (Community) pandemic management

**
a. Administrative level:
**

- Overall Information about OPD, In-Patient Department (IPD) and other facilities at district level
- Do you have pandemic preparedness plan for district?
- Did the district use any protocol or guidelines for clinical management of H1N1?
- Decision-makers, process of decision-making, communication with public health system, communication from public health system after declaring Phase 6: Pandemic phase
- Was there any spokesperson deputed at district level? Roles and responsibilities of the spokesperson? Media management/public relation officer/management
- Whether any sensitization/orientation of clinicians, supporting staff took place?
- How was public health staff sensitized?
- Safety and precautionary measures used at district hospital level: vaccination, use of N-95 Masks, etc
- Reporting to District Collector, state in charge: frequency, type of reports

**b. Patients Management:**
b.1. At OPD level:

- Throat swabs: testing facilities, available treatment, number of staff, inter departmental co-ordination
- Profile of patients: area, symptoms, age group, class
- Challenges in differentiating patients with swine flu and other ILIs
- Use of Tamiflu: prevention [Prophylaxis] and/or treatment and side effects. Your view about ‘Tamiflu’
- Vaccine: When was it became available, information about efficacy of the vaccine, which kind of vaccine [Availability, manufacturers, cost, who were vaccinated on priority, side effects of vaccine for general public as well as pregnant women, for people with chronic illnesses like diabetes etc]
- Vaccine uptake during the pandemic situation
- Your views about swine flu vaccine [applicability, effectiveness, herd immunity] Challenges faced at OPD level: Heavy patient load, counseling the patients

b.2. At IPD level:

- Profile of patients: Area, symptoms, age group, class
- Isolation ward, Facilities at ICU, ventilators, number of beds, Availability of drugs, instruments, PPE
- How was additional beds/additional manpower arranged?
- Staff arrangement, staff training
- Ambulance service
- How many deaths happened due to swine flu: Profile of the death cases, complication observed in the cases, could those deaths have prevented?
- Post Mortem services: Challenges
- Biological waste material management:
- Challenges faced at OPD levels: expert manpower, infrastructure, handling patients and family members
- Steps taken by district in managing community level panic: Awareness programmes
- What role media played during the pandemic situation: Awareness creation, creating panic?
- Post pandemic Vaccine Use and Efficacy
- Key lessons for future preparedness
- For district: comparative picture: pandemic situation, today and steps for future
- For public health system
- For private medical institutions
- In clinical management of pandemic illnesses
- Media’s role
- Community’s role
- Medical associations’ role
- Role of non-allopath, etc

## Appendix 2

Appendix 2. Interview Guide for District Policy-Makers and Administrators

1. Qualification: Training and specialization, position(s) held
2. Description about course and development of 2009 H1N1 influenza pandemic situation
3. How the response strategy was planned at district level?
• Clinical management and public health measures employed for pandemic control
• Communication with public health system, communication from public health system after declaring Phase 6: Pandemic phase
• Need for evoking Epidemic Act
• Your role during the pandemic management
• Actors involved in planning the response
• Actors involved in implementing the response
• Coordination between centre, state and district
• Role of media in pandemic management
4. Your reflections on key successes and opportunities for improvement and financial Implications
5. Key lessons and steps taken for future preparedness
• Steps for future preparedness including inter-pandemic activities regarding:
• District Administration
• For health system
• Media’s role
• Community’s role
