# Supplementary material for: Public Health Policy and Experience of the 2009 H1N1 Influenza Pandemic in Pune, India
Source: Int J Health Policy Manag. 2017 May 9;7(2):154–66. doi: 10.15171/ijhpm.2017.54 (PMC5819375; doi:10.15171/ijhpm.2017.54)
Supplement: Supplementary file — A supplementary file has been added for the “Contingency Plan for Management of Influenza A (H1N1),” published by the Directorate of Health Services, Government of Maharashtra. This document was accessed and downloaded for analysis on July 14, 2014 from the link cited in the document: http://maha-arogya.gov.in. This link, however, is no longer active, and we provide the document as a supplementary file. [file ijhpm-7-154-s001.pdf]

CONTINGENCY PLAN

FOR MANAGEMENT OF

INFLUENZA A (H1N1)

http://maha-arogya.gov.in

JUNE 2009
DIRECTORATE OF HEALTH SERVICES,
GOVERNMENT OF MAHARASHTRA

CONTINGENCY PLAN FOR MANAGEMENT OF INFLUENZA A (H1N1)

INTRODUCTION:-

The world is getting affected by emergence of Influenza A (H1N1) virus that might have originated either in swine and then shifted from swine to humans or due to a super-infection in humans by an unknown variant with variable genetic mix. Influenza (Flu) pandemics are caused by new influenza viruses that have recently adapted to humans and resemble major natural disasters both in terms of recurrence and magnitude. The influenza virus, known to be circulating as a pathogen in the human population since at least the 16th century is notable for its unique ability to cause recurrent epidemics and global pandemics. Genetic re-assortments in the influenza virus cause fast and unpredictable antigenic changes in important immune targets leading to recurrent epidemics of febrile respiratory disease every 1 to 3 years consistently necessitated the development of new vaccines. Each century has seen some pandemics rapidly progressing to all parts of the world due to emergence of a novel virus to which the overall population holds no immunity. Historically incidence of Influenza appears in cyclical pattern emerging after every 50 years. The last two incidences of severe outbreak were in 1918-19 and 1957-58. These outbreaks lead to large number of deaths. A fear voiced by WHO is surge of H1N1 virus might recur in about 3 month's time with greater virulence. In case such a pandemic occurs, it may affect almost 70 percent of workforce. It is of paramount importance to contain the infection at this stage so as to avert the impending calamity.

OBJECTIVES:-

1. Surveillance of influenza like illnesses (ILI)
2. Early detection and their management.
3. Containment of the transmission of infection.
4. Decrease of social disruption and economic loss.
5. Maintenance of business continuity beyond health sectors.

ACTION PLAN:-

1. **INSTITUTIONAL FRAMEWORK-**

1.1 As WHO has declared phase 6 of pandemic alert about Influenza A (H1N1), state crisis management group has decided to activate its contingency plan. **(Refer Government Resolution dated 11 June 2009, No: Swine Flu 2009/148/CR122/09/PH-5)**

1.2 Joint Director,(Malaria,Filaria & Waterborne Diseases),Pune is identified nodal authority for managing Influenza A (H1N1) control activities in the state.

http://maha-arogya.gov.in

1.3 State and District Crisis Management Groups are formed for business continuity in non health sectors, as per Government Resolution dated 11 June 2009, No: Swine Flu 2009/148/CR122/09/PH-5.

1.4 The **business continuity planning is interlinked** and is required to be build up with following assumptions in mind:

i) Expected absenteeism may go up to 70%.

ii) Every service provider needs to be assured that other services in their optimal set up are available.

iii). Financial institutions remain operational.

iv) Decision makers will follow chain of command to ensure continuity & to avoid delays and associated panic.

v) Ensure availability of essential products.

1.5 **State / District Level Operations**

a. Information from all critical sites will be received by the nodal officer of district Crisis Management Group followed by analysis of the factual information and an action taken report (inclusive of future requirements) will be sent to state crisis management group

b. The information will be analyzed critically and checked whether it has compiled all the vital issues along with any relevant information pertaining to local factors and eventually a weekly status report of essential service provisions will be prepared.

c. The report will enumerate the following:

i. Various limitations to both manpower and resources and recommendation to utilize optimal contingencies to meet the enhanced demands;

ii Future actions which will be undertaken to meet the demands (specifying cooperation/ actions of any linked department / Ministry, if required).

d. These reports will also be sent to MoH&FW, NCMC, NDMA and all the departments concerned at central level.

1.6. State and District Crisis Management Groups will ensure workplace and Personnel resilience as per the guidelines of National Disaster Management Authority (NDMA), New Delhi.

http://maha-arogya.gov.in

2. **Surveillance –**

2.1 **Control Room** – has been started from 28th April 2009, at Directorate of Health Services to monitor & co-ordinate the activities of Influenza A (H1N1) control.(Phone-022-22653460).

2.2 **Screening** of Visitors coming from affected countries has been started at the International Airport / Ports in Mumbai and International Airport in Pune & Nagpur from 27th June 2009.

Sr. No	Places	Date of Commencement of Screening	Doctors	Paramedical Workers
1	Airport, Mumbai	30.4.2009	30	4
2	Airport,Pune	2.04.2009	3	3
3	Airport,Nagpur	4.05.2009	3	2
4	Mumbai Port Trust	29.04.2009	18	6
5	JNPT	29.04.2009	3	4

2.3 **Isolation of Suspected Cases-** Three infectious disease control Hospitals has been identified as the sentinel centers in the state, for isolation and laboratory sample collection of the suspected cases-

i) Kasturba Hospital, Mumbai.
ii) Naidu Hospital,Pune.
iii) Government Medical College, Nagpur.

2.4 **Active and Passive Surveillance of Influenza Like Illness (ILI)**- After raising pandemic alert from phase 5 to 6 by WHO, all District Health Officers & Civil Surgeons are asked to start surveillance of ILI in their districts on war footing and submit their daily reports to nodal authority through their respective Deputy Directors. State multi-disciplinary Rapid Response Team (RRT) will do active surveillance in the districts of the state bordering with Hyderabad and Goa, from where positive cases have already been reported.

2.5 The RRT would identify the high risk groups for administration of chemoprophylaxis. The RRT will identify and train manpower for laboratory sample collection of suspected cases.

2.6 District health authorities will ensure collection of samples whenever necessary and transport it to NIV,Pune with due precaution.

3. **Clinical Management**:-

3.1. Isolation ward of minimum 5 beds will be made available at every District Hospital of the state to isolate and treat the probable/suspect/confirmed cases.

.

3.2 The cases would be managed in identified hospitals having isolation facilities that stringently follow standard infection control practices.

http://maha-arogya.gov.in

3.3 District health authorities would identify suspected/confirmed cases using the standard case definition.

3.4 District administration would ensure transport of such cases from affected areas to the designated hospital.

3.5 All cases should be treated according to guidelines issued by ministry of Health and Family Welfare, GOI, New Delhi.

3.6. Nodal authority would ensure adequate supply of all necessary drugs and required equipments to all identified hospitals.

4. PUBLIC HEALTH MEASURES :

4.1 The guideline for use of Personal Protective Equipments should be given to all medical, nursing and paramedical personnel.

4.2 The contact cases would be quarantined in-house.

4.3 School children suffering from ILI would be asked for home quarantine for minimum 7 days or 24 hours after symptoms have reduced completely, whichever is longer.

4.4 Intensive IEC campaign would be undertaken in the high risk areas. Generic IEC guidelines do's and don'ts and FAQs would be disseminated among the masses.

5. Logistics and Supply:

5.1 Suitable stocks of anti-viral drugs for treatment as well as chemoprophylaxis, PPE and critical care equipments are stockpiled at Regional director, Health and Family Welfare, GOI, Pune.

Balance Of Oseltamivir capsules in the State

Sr. No.	Name Of The Institution	Balance
1	Reginal Director,Health & Family Welfare,GOI,Pune	19200
2	JD,(Malaria,Filaria,Waterborne Diseaes),Pune	3980
3	Govt.MedicalCollege,Nagpur	500
4	Dr.Naidy Infectious Diseases Hospital,Pune	100
5	Sir J.J.Medical College & Hospital,Mumbai	1000
6	Brihanmumbai Muncipal Corporation,Mumbai	1000

http://maha-arogya.gov.in

5.2 State Nodal authority would make available sufficient stocks of anti-viral drugs at all district head quarters and especially to districts bordering to Hyderabad and Goa on priority basis.

5.3 State Nodal Authority would mobilize additional resources in terms of manpower and material to the high priority area or wherever required.

6. ORIENTATION OF HUMAN RESOURSES-

6.1 State RRT would arrange for divisional and district Influenza A (H1N1) Orientation workshops for medical and paramedical staff of government and non government sectors to enable them to face any kind of eventuality.

6.2 Departments providing essential services would orient their staffs with do's and don'ts regarding containment of the disease and FAQs about the disease.

7. PARTICIPATION OF NGOs :

There are dedicated NGOs existing in health as well as non health sectors in our state. All these organizations must register with district and/or respective state authorities so that their services are utilized and duties delegated for the appropriate charter which depends on the strengths that they possess in a particular field.

8. COMMUNICATIONS AND MEDIA MANAGEMENT:

8.1 State Nodal Authority and Influenza A Control Room DHS would communicate to all concerned departments regarding the status and other information related to Influenza A. Directorate of health services would maintain a web based interactive public information system.

8.2 The Chairman, State Crisis Management Group or any officer designated by him/her would address the media.

8.3 Support of Department of Telecommunications would be sought in areas without landline or cellular connectivity.

9. MISCLLANEOUS:

The contingency plan would be circulated to all concerned. This would include contact numbers of all important officers.

http://maha-arogya.gov.in

Influenza A (H1N1)

Clinical management Protocol
and
Infection Control Guidelines

http://maha-arogya.gov.in

Directorate of Health Services
Government of Maharashtra

INTRODUCTION:-

The world is getting affected by emergence of Influenza A (H1N1) virus that might have originated either in swine and then shifted from swine to humans or due to a super-infection in humans by an unknown variant with variable genetic mix. Influenza (Flu) pandemics are caused by new influenza viruses that have recently adapted to humans and resemble major natural disasters both in terms of recurrence and magnitude. The influenza virus, known to be circulating as a pathogen in the human population since at least the 16th century is notable for its unique ability to cause recurrent epidemics and global pandemics. Genetic re-assortments in the influenza virus cause fast and unpredictable antigenic changes in important immune targets leading to recurrent epidemics of febrile respiratory disease every 1 to 3 years consistently necessitated the development of new vaccines. Each century has seen some pandemics rapidly progressing to all parts of the world due to emergence of a novel virus to which the overall population holds no immunity.
Historically incidence of Influenza appears in cyclical pattern emerging after every 50 years. The last two incidences of severe outbreak were in 1918-19 and 1957-58. These outbreaks lead to large number of deaths. A fear voiced by WHO is surge of H1N1 virus might recur in about 3 month’s time with greater virulence. In case such a pandemic occurs, it may affect almost 70 percent of workforce.

SWINE FLU IN PIGS:-

Swine Influenza (swine flu) is a respiratory disease of pigs caused by type A influenza virus that regularly causes outbreaks of influenza in pigs. Swine flu viruses cause high levels of illness and low death rates in pigs. Swine influenza viruses usually circulate among swine throughout the year, but most outbreaks occur during the late fall and winter months similar to outbreaks in humans. The classical swine flu virus (an influenza type A H1N1 virus) was first isolated from a pig in 1930. H3N2 influenza viruses began circulating among pigs from 1998. The H3N2 viruses initially were introduced into the pig population from humans. Like all influenza viruses, swine flu viruses change constantly. Pigs can be infected by avian influenza, human influenza viruses as well as swine influenza viruses and hence the pigs are known to be a mixing vessel. When influenza viruses from different species infect pigs, the viruses can re-assort (i.e. swap genes) and new viruses , a mix of swine, human and/or avian influenza viruses - can emerge leading to development of new novel strain for which human beings do not have no immunity. There are four main influenza type A virus subtypes that have been isolated in pigs: H1N1, H1N2, H3N1 and H3N2. However, most of the recently isolated influenza viruses from pigs have been H1N1 viruses. Swine flu virus spreads mostly through close contact among pigs and possibly from contaminated objects moving between infected and uninfected pigs. Symptoms of swine flu in pigs can include sudden onset of fever, depression, coughing (barking), discharge from the nose or eyes, sneezing, breathing difficulties, eye redness or inflammation, and going off feed.

http://maha-arogya.gov.in

Influenza A (H1N1) IN HUMANS:-

Influenza A (H1N1)viruses do not normally infect humans. However, sporadic human infections with swine flu have occurred. Most commonly, these cases occur in persons having direct exposure to pigs. In addition, there have been sporadic cases of one person spreading Influenza A (H1N1) to others. Occasional human Influenza A (H1N1)virus infection occurs every one to two years in the U.S., but from December 2005 through February 2009, 12 cases of human infection with Influenza A (H1N1) have been reported.

INFLUENZA A (H1N1) OUTBREAK:

Recently, human cases of swine influenza A (H1N1) virus infection have been reported in several countries. This is a novel influenza A virus that has not been identified in people before, and human-to-human transmission of the virus appears to be ongoing and thus represents a real pandemic threat. WHO has upgraded the phasing of pandemic influenza from Phase -3 to Phase 5 on 29th April 2009 and to phase 6 on 11th June 2009.

Current Situation:

The current situation regarding the outbreak of influenza A (H1N1) is evolving rapidly. As on 15 June 2009, 76 countries have officially reported 35,928 confirmed cases of influenza A(H1N1) infection with 163 deaths. Of these, India has reported 30 cases so far without any death. United States has reported 13,217 laboratory confirmed human cases, including 27 deaths. Mexico has reported 6241 confirmed human cases including 108 deaths.

The following countries have reported laboratory confirmed cases with deaths –

Countries	Total Deaths
**Mexico**	**108**
**USA**	**45**
**Canada**	**4**
**Chile**	**2**
**Columbia**	**1**
**Costa Rica**	**1**
**Dominican Republic**	**1**
**Gautemala**	**1**

Unlike the experience in Mexico, the United States is currently reporting infection by the identical virus strain or less severe clinical spectrum of disease. Mexican health officials have reported several hundred suspect cases, including several deaths associated with swine influenza A (H1N1) virus infection. In Mexico, many patients have experienced rapidly progressive pneumonia, respiratory failure and acute respiratory distress syndrome (ARDS) requiring mechanical ventilation.

http://maha-arogya.gov.in

India:

By 15/06/09, samples 0f 318 persons have been tested of which 30 have been tested positive for novel influenza A (H1N1).Of these, two are indigenous cases who got the infection from the positive cases traveled from abroad.

State wise break up of confirmed H1N1 cases

S.N.	States	Confirmed Influenza A (H1N1) cases
1	Andhra Pradesh	12
2	Punjab	7
3	Delhi	6
4	Karnataka	2
5	Tamilnadu	2
6	Goa	1
	Total	30

Maharashtra:-

By 15/06/09, samples of 23 persons have been tested. All these samples proved to be negative for novel influenza A (H1N1).

INCUBATION PERIOD:-

1-7 Days

Symptoms

Manifestations of H1N1 influenza (swine flu) are similar to those of seasonal influenza. Patients present with symptoms of acute respiratory illness, including at least 2 of the following:

- Fever
- Cough
- Sore throat
- Body aches
- Headache
- Chills and fatigue
- Diarrhea and vomiting (possible)

http://mana-arogya.gov.in

Persons with these symptoms should call their health care provider promptly.

In children, signs of severe disease include apnea, tachypnea, dyspnea, cyanosis, dehydration, altered mental status, and extreme irritability.

Infectious period-

The infectious period for a confirmed case is defined as 1 day prior to the onset of symptoms to 7 days after onset.

TRANSMISSION:

- Influenza viruses can be directly transmitted from pigs to people and from people to pigs.

- Human infection with flu viruses from pigs are most likely to occur when people are in close proximity to infected pigs, such as in pig barns and livestock exhibits housing pigs at fairs.

- Human-to-human transmission of influenza A (H1N1) can also occur. At present, in India we have 2 indigenous cases This is thought to occur in the same way as seasonal flu which is mainly person-to person transmission through coughing or sneezing by people infected with the influenza virus.

- Disease spreads very quickly among the population especially in crowded places.

- Cold and dry weather enables the virus to survive longer outside the body than in other conditions and, as a consequence, seasonal epidemics in temperate areas appear in winter.

- People may become infected by touching/handling something contaminated with flu viruses on it and then touching their mouth or nose.

- Influenza A (H1N1) viruses are not transmitted by food.

- Eating properly handled and cooked pork (at an internal temperature of ≥160°F) and pork products is safe.

DIAGNOSIS OF INFLUENZA A (H1N1):

For diagnosis of influenza A (H1N1) infection, respiratory specimen would generally need tobe collected within the first 4 to 5 days of illness (when an infected person is most likely to be shedding virus). However, some persons, especially children, may shed virus for 10 days or longer.

http://nrhm-aroogya.gov.in

Treatment Recommendations

Treatment is largely supportive and consists of bed rest, increased fluid consumption, cough suppressants, and antipyretics and analgesics (eg, acetaminophen, non steroidal anti-inflammatory drugs) for fever and myalgias. Severe cases may require intravenous hydration and other supportive measures. Antiviral agents may also be considered for treatment or prophylaxis (see Medications).

Patients should be encouraged to stay home if they become ill, to avoid close contact with people who are sick, to wash their hands often, and to avoid touching their eyes, nose, and mouth. The CDC recommends the following actions when human infection with H1N1 influenza (swine flu) is confirmed in a community:

Home isolation

- Patients who develop flulike illness (ie, fever with either cough or sore throat) should be strongly encouraged to self-isolate in their home for 7 days after the onset of illness or at least 24 hours after symptoms have resolved, whichever is longer.
- Patients who have difficulty breathing or shortness of breath or who are believed to be severely ill should seek immediate medical attention.
- If the patient must go into the community (eg, to seek medical care), he or she should wear a face mask to reduce the risk of spreading the virus in the community when coughing, sneezing, talking, or breathing. If a face mask is unavailable, ill persons who need to go into the community should use tissues to cover their mouth and nose while coughing.
- While in home isolation, patients and other household members should be given infection control instructions, including frequent hand washing with soap and water. Use alcohol-based hand gels (containing at least 60% alcohol) when soap and water are not available and hands are not visibly dirty. Patients with H1N1 influenza should wear a face mask when within 6 feet of others at home.

Household contacts who are not ill

- Remain home at the earliest sign of illness.
- Minimize contact in the community to the extent possible.
- Designate a single household family member as caregiver for the patient to minimize interactions with asymptomatic persons.

School dismissal and childcare facility closure

- Strong consideration should be given to close schools upon a confirmed case of H1N1 flu or a suspected case epidemiologically linked to a confirmed case.
- Cancellation of all school or childcare related gatherings should also be announced.
- Encourage parents and students to avoid congregating outside of the school if school is canceled.
- Duration of schools and childcare facilities closings should be evaluated on an ongoing basis depending on epidemiological findings.

http://mahaarogya.gov.in

- Consultation with local or state health departments is essential for guidance concerning when to reopen schools. If no additional confirmed or suspected cases are identified among students (or school-based personnel) for a period of 7 days, schools may consider reopening.
- Schools and childcare facilities in unaffected areas should begin preparation for possible school closure.

Social distancing

- Large gatherings linked to settings or institutions with laboratory-confirmed cases should be canceled (eg, sporting events or concerts linked to a school with cases); other large gatherings in the community may not need to be canceled at this time.
- Persons with underlying medical conditions who are at high risk for complications of influenza should consider avoiding large gatherings.

Medications

Laboratory testing has found the H1N1 influenza A virus susceptible to the prescription antiviral drugs oseltamivir (Tamiflu) and zanamivir (Relenza).

The usual vaccine for influenza administered at the beginning of the flu season is not effective for this viral strain. Also, other antiviral agents (eg, amantadine, rimantadine) are not recommended because of recent resistance to other influenza strains documented over the past several years.

Basic supportive care (ie, hydration, analgesics, cough suppressants) should be prescribed. Empiric antiviral treatment should be considered for confirmed, probable, or suspected cases of H1N1 influenza. Treatment of hospitalized patients and patients at higher risk for influenza complications should be prioritized.

The recommended duration of treatment is 5 days.

- Prophylaxis should be provided till 10 days after last exposure (maximum period of 6 weeks)

- Prophylaxis with antiviral agents should also be considered in the following individuals (pre-exposure or post exposure):
  - Close household contacts of a confirmed or suspected case who are at high risk for complications (e g, chronic medical conditions, persons >65 y or <5 y, pregnant women)
  - School children at high risk for complications who have been in close contact with a confirmed or suspected case

http://maha-arogya.gov.in

    - Travelers to affected countries who are at high risk for complications (eg, chronic medical conditions, persons >65 y or <5 y, pregnant women)
    - Health care providers or public health workers who were not using appropriate personal protective equipment during close contact with a confirmed or suspected case
- Pre-exposure prophylaxis can be considered in the following persons:
    - Any health care provider who is at high risk for complications (eg, persons with chronic medical conditions, adults >65 y, pregnant women)
    - Individuals not considered to be at high risk but who are nonetheless traveling to affected countries, first responders, or border workers who are working in areas with confirmed cases

Antiviral Agents

Drugs indicated for treatment of H1N1 influenza A virus include neuraminidase inhibitors (ie, oseltamivir and zanamivir).

Oseltamivir (Tamiflu)

Duration of administration for treatment is 5 days. Post exposure prophylaxis should be initiated within 7 d of exposure and continued for at least 10 days. Pre-exposure prophylaxis should be initiated during potential exposure period and continue for 10 days after last known exposure.

Oseltamivir is available as 30-mg, 45-mg, and 75-mg oral capsules and as a powder for suspension that contains 12 mg/mL after reconstitution.

- **Adult dose**
    - Treatment for acute illness: 75 mg PO bid for 5 d
    - Prophylaxis: 75 mg PO (Please refer to duration of prophylaxis specific for post exposure.)
- **Pediatric dose**
    - Treatment for acute illness and age <1 year
        - <3 months: 12 mg PO bid for 5 d
        - 3-5 months: 20 mg PO bid for 5 d
        - 6-11 months: 25 mg PO bid for 5 d
    - Treatment for acute illness and age >1 year
        - <15 kg: 30 mg PO bid for 5 d
        - 15-23 kg: 45 mg PO bid for 5 d
        - 23-40 kg: 60 mg PO bid for 5 d
        - >40 kg: Administer as in adults
    - Prophylaxis and age <1 year
        - <3 months: Data limited; not recommended unless situation judged critical
        - 3-5 months: 20 mg PO qd
        - 6-11 months: 25 mg PO qd
    - Prophylaxis and age >1 year

http://maha-arogya.gov.in

- <15 kg: 30 mg PO qd
- 15-23 kg: 45 mg PO qd
- 23-40 kg: 60 mg PO qd
- >40 kg: Administer as in adults

Zanamivir (Relenza)

. Zanamivir is effective against both influenza A and B. The preparation of zanamivir is in powder form for inhalation via the Diskhaler oral inhalation device. Circular foil discs that contain 5-mg blisters of drug are inserted into the supplied inhalation device. Individuals with asthma or other respiratory conditions that may decrease ability to inhale drug should be given oseltamivir.

Additional pediatric considerations

Aspirin or aspirin-containing products (eg, bismuth subsalicylate [Pepto Bismol]) should not be included in the treatment of confirmed or suspected viral infection in persons aged 18 years or younger because of the risk of Reye syndrome. For relief of fever, other antipyretic medications (eg, acetaminophen, nonsteroidal anti-inflammatory drugs) are recommended.

Pregnant women

Oseltamivir and zanamivir are "Pregnancy Category C" medications, indicating that no clinical studies have been conducted to assess the safety of these medications in pregnant women.

Because of the unknown effects of influenza antiviral drugs on pregnant women and their fetuses, oseltamivir or zanamivir should be used during pregnancy only if the potential benefit justifies the potential risk to the embryo or fetus; the manufacturers' package inserts should be consulted. However, no adverse effects have been reported among women who received seltamivir or zanamivir during pregnancy or among infants born to women who have received oseltamivir or zanamivir.

Pregnancy should not be considered a contraindication to oseltamivir or zanamivir use. Because zanamivir is an inhaled medication and has less systemic absorption, some experts prefer zanamivir over oseltamivir for use in pregnant women, when feasible.

PREVENTIVE MEASURES:-

Do's and Dont's for the public:

Do's

- Cover your mouth and nose with a handkerchief or tissue while coughing or sneezing.
- Wash your hands every time after coughing or sneezing, thoroughly with soap and water, before and after touching your nose, eyes or mouth.
- Avoid crowded places.

http://maha-arogya.gov.in

• Stay at home if infected with flu like illness.
• Keep at least an arm's distance from people affected with symptoms of influenza like cough, running nose, sneezing and fever.
• Sleep well, stay physically active and effectively manage stress.
• Drink plenty of water and eat nutritious food.

Dont's

• Shake hands, hug and kiss socially, or use other contact greetings.
• Take medicines without consulting the physician.
• Spit outdoors.
• Aspirin not to be used in children.

Standard Operating Procedures on Use of PPE

Personal Protection Equipments

PPE reduces the risk of infection if used correctly. It includes:
• Gloves (nonsterile),
• Mask (high-efficiency mask) / Three layered surgical mask,
• Long-sleeved cuffed gown,
• Protective eyewear (goggles/visors/face shields),
• Cap (may be used in high risk situations where there may be increased aerosols),
• Plastic apron if splashing of blood, body fluids, excretions and secretions is anticipated.

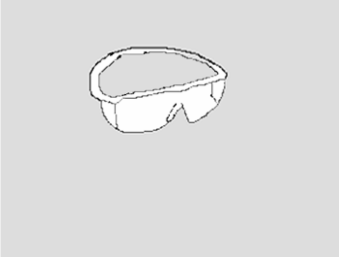

Goggles

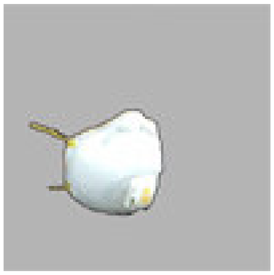

N-95 Mask

OR

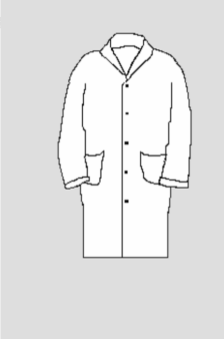

Gown(must for lab work)

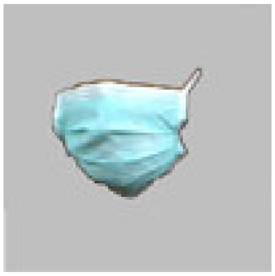

Triple layer Mask

http://[illegible]arogya.gov.in

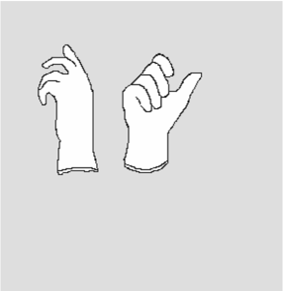

Gloves

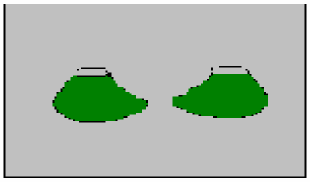

Shoe covers

The PPE should be used in situations were regular work practice requires unavoidable, relatively closed contact with the suspected human case.

Correct procedure for applying PPE in the following order:

1. Follow thorough hand wash
2. Wear the coverall.
3. Wear the goggles/ shoe cover/and head cover in that order.
4. Wear face mask
5. Wear gloves

The masks should be changed after every six to eight hours.

Remove PPE in the following order:

- Remove gown (place in rubbish bin).
- Remove gloves (peel from hand and discard into rubbish bin).
- Use alcohol-based hand-rub or wash hands with soap and water.
- Remove cap and face shield (place cap in bin and if reusable place face shield in container for decontamination).
- Remove mask - **by grasping elastic behind ears – do not touch front of mask**
- Use alcohol-based hand-rub or wash hands with soap and water.
- Leave the room.
- Once outside room use alcohol hand-rub again or wash hands with soap and water.

Used PPE should be handled as waste as per waste management protocol

1.1 Hand Hygiene

Hand hygiene is the single most important measure to reduce the risk of transmitting infectious organism from one person to other.

Hands should be washed frequently with soap and water / alcohol based hand rubs/ antiseptic hand wash and thoroughly dried preferably using disposable tissue/ paper/ towel.

http://maha-arogya.gov.in

- After contact with respiratory secretions or such contaminated surfaces.
- Any activity that involves hand to face contact such as eating/ normal grooming / smoking etc.

Steps of hand washing

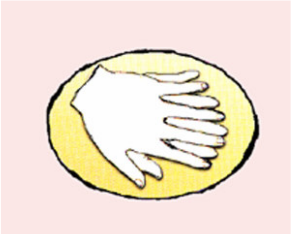

Step 1.
Wash palms and fingers.

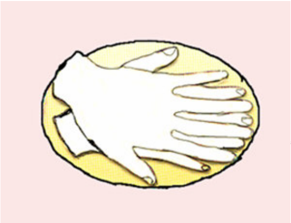

Step 2.
Wash back of hands.

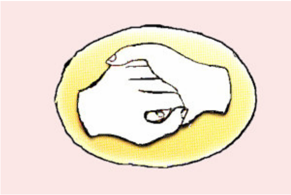

Step 3.
Wash fingers and knuckles.

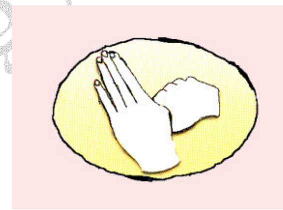

Step 4.
Wash thumbs.

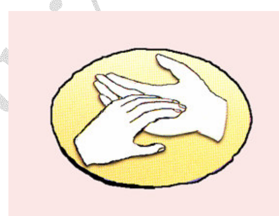

Step 5.
Wash fingertips.

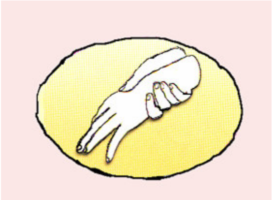

Step 6.
Wash wrists.

http://maha-arogya.gov.in

1.2 Respiratory Hygiene/Cough Etiquette

The following measures to contain respiratory secretions are recommended for all individuals with signs and symptoms of a respiratory infection.

- ♦ Cover the nose/mouth with a handkerchief/ tissue paper when coughing or sneezing;
- ♦ Use tissues to contain respiratory secretions and dispose of them in the nearest waste receptacle after use;
- ♦ Perform hand hygiene (e.g., hand washing with non-antimicrobial soap and water, alcohol-based hand rub, or antiseptic hand wash) after having contact with respiratory secretions and contaminated objects/materials

1.3 Staying away

❋ Stay at least six feet away from a person having cough or sneeze.

1.4 Use of mask

Masks are not recommended for individuals or community. As a matter of abundant precaution, confirmed/ suspected cases managed at home and there family contacts are asked to use three layered surgical masks.

2. Infection control measures at health facility

2.1 Droplet Precautions:

Advise healthcare personnel to observe Droplet Precautions (i.e., wearing a surgical or procedure masks for close contact), in addition to Standard Precautions, when examining a patient with symptoms of a respiratory infection, particularly if fever is present. These precautions should be maintained until it is determined that the cause of symptoms is not an infectious agent that requires Droplet Precautions.

2.2 Visual Alerts

Post visual alerts (in appropriate languages) at the entrance to outpatient facilities (e.g., emergency departments, physician offices, outpatient, clinics) instructing patients and persons who accompany them (e.g., family, friends) to inform healthcare personnel of symptoms of a respiratory infection when they first register or care and to **practice Respiratory Hygiene/Cough Etiquette.**

2.3 **Use of PPE**

- o The medical, nurses and paramedics attending the suspect/ probable / confirmed case should wear full complement of PPE (Annexure-IX).
- o Use N-95 masks during aerosol-generating procedures.
- o Perform hand hygiene before and after patient contact and following contact with contaminated items, whether or not gloves are worn.
- o Sample collection and packing should be done under full cover of PPE.

2.4 **Decontaminating contaminated surfaces, fomites and equipments**

Cleaning followed by disinfection should be done for contaminated surfaces and equipments.

- o use phenolic disinfectants, quaternary ammonia compounds , alcohol or sodium hypochlorite. Patient rooms/areas should be cleaned at least daily and

http://maha-arogya.gov.in

terminally after discharge. In addition to daily cleaning of floors and other horizontal surfaces, special attention should be given to cleaning and disinfecting frequently touched surfaces.

- To avoid possible aerosolization of AI virus, damp sweeping should be performed.
- Clean heavily soiled equipment and then apply a disinfectant effective against influenza virus before removing it from the isolation room/area.
- When transporting contaminated patient-care equipment outside the isolation room/area, use gloves followed by hand hygiene. Use standard precautions and follow current recommendations for cleaning and disinfection or sterilization of reusable patient-care equipment.

2.5 Guidelines for waste disposal

- All the waste has to be treated as infectious waste and decontaminated as per standard procedures
- Articles like swabs/gauges etc are to be discarded in the Yellow coloured autoclavable biosafety bags after use, the bags are to be autoclaved followed by incineration of the contents of the bag.
- Waste like used gloves, face masks and disposable syringes etc are to be discarded in Blue/White autoclavable biosafety bags which should be autocalaved/microwaved before disposal
- All hospitals and laboratory personnel should follow the standard guidelines (Biomedical waste management and handling rules, 1998) for waste management.

FREQUENTLY ASKED QUESTIONS:

What can I do to protect myself from catching influenza A(H1N1)?

The main route of transmission of the new influenza A(H1N1) virus seems to be similar to seasonal influenza, via droplets that are expelled by speaking, sneezing or coughing. You can prevent getting infected by avoiding close contact with people who show influenza-like symptoms (trying to maintain a distance of about 1 metre if possible) and taking the following measures:

- avoid touching your mouth and nose;
- clean hands thoroughly with soap and water, or cleanse them with an alcohol-based hand rub on a regular basis (especially if touching the mouth and nose, or surfaces that are potentially contaminated);
- avoid close contact with people who might be ill;
- reduce the time spent in crowded settings if possible;
- improve airflow in your living space by opening windows;
- practise good health habits including adequate sleep, eating nutritious food, and keeping physically active.

http://maha-arogya.gov.in

What about using a mask? What does WHO recommend?

If you are not sick you do not have to wear a mask.

If you are caring for a sick person, you can wear a mask when you are in close contact with the ill person and dispose of it immediately after contact, and cleanse your hands thoroughly afterwards.

If you are sick and must travel or be around others, cover your mouth and nose.

Using a mask correctly in all situations is essential. Incorrect use actually increases the chance of spreading infection.

How do I know if I have influenza A(H1N1)?

You will not be able to tell the difference between seasonal flu and influenza A(H1N1) without medical help. Typical symptoms to watch for are similar to seasonal viruses and include fever, cough, headache, body aches, sore throat and runny nose. Only your medical practitioner and local health authority can confirm a case of influenza A(H1N1).

What should I do if I think I have the illness?

If you feel unwell, have high fever, cough or sore throat:

- stay at home and keep away from work, school or crowds;
- rest and take plenty of fluids;
- cover your nose and mouth when coughing and sneezing and, if using tissues, make sure you dispose of them carefully. Clean your hands immediately after with soap and water or cleanse them with an alcohol-based hand rub;
- if you do not have a tissue close by when you cough or sneeze, cover your mouth as much as possible with the crook of your elbow;
- use a mask to help you contain the spread of droplets when you are around others, but be sure to do so correctly;
- inform family and friends about your illness and try to avoid contact with other people;
- If possible, contact a health professional before traveling to a health facility to discuss whether a medical examination is necessary.

Should I take an antiviral now just in case I catch the new virus?

No. You should only take an antiviral, such as oseltamivir or zanamivir, if your health care provider advises you to do so. Individuals should not buy medicines to prevent or fight this new influenza without a prescription, and they should exercise caution in buying antivirals over the Internet.

What about breastfeeding? Should I stop if I am ill?

No, not unless your health care provider advises it. Studies on other influenza infections show that breastfeeding is most likely protective for babies - it passes on helpful maternal immunities and lowers the risk of respiratory disease.

http://mahi-arogya.gov.in

Breastfeeding provides the best overall nutrition for babies and increases their defense factors to fight illness.

When should someone seek medical care?

A person should seek medical care if they experience shortness of breath or difficulty breathing, or if a fever continues more than three days. For parents with a young child who is ill, seek medical care if a child has fast or labored breathing, continuing fever or convulsions (seizures).

Supportive care at home - resting, drinking plenty of fluids and using a pain reliever for aches - is adequate for recovery in most cases. (A non-aspirin pain reliever should be used by children and young adults because of the risk of Reye's syndrome.)

Should I go to work if I have the flu but am feeling OK?

No. Whether you have influenza A(H1N1) or a seasonal influenza, you should stay home and away from work through the duration of your symptoms. This is a precaution that can protect your work colleagues and others.

Can I travel?

If you are feeling unwell or have symptoms of influenza, you should not travel. If you have any doubts about your health, you should check with your health care provider.

What is phase 6?

Phase 6 is a pandemic, according to the WHO definition.

Phase 1-3	Predominantly animal infection, few human infections
Phase 4	Sustained human to human transmission
Phase 5-6	Widespread human infection
POST PEAK	Possibility of recurrent events
POST PANDEMIC	Disease activity at seasonal levels

What about severity?

At this time, WHO considers the overall severity of the influenza pandemic to be moderate. This assessment is based on scientific evidence available to WHO, as well as input from its Member States on the pandemic's impact on their health systems, and their social and economic functioning.

The moderate assessment reflects that:

1. Most people recover from infection without the need for hospitalization or medical care.

http://maha-arogya.gov.in

2. Overall, national levels of severe illness from influenza A(H1N1) appear similar to levels seen during local seasonal influenza periods, although high levels of disease have occurred in some local areas and institutions.
3. Overall, hospitals and health care systems in most countries have been able to cope with the numbers of people seeking care, although some facilities and systems have been stressed in some localities.

WHO is concerned about current patterns of serious cases and deaths that are occurring primarily among young persons, including the previously healthy and those with pre-existing medical conditions or pregnancy.

Large outbreaks of disease have not yet been reported in many countries, and the full clinical spectrum of disease is not yet known.

Does WHO expect the severity of the pandemic to change over time?

The severity of pandemics can change over time and differ by location or population.

Close monitoring of the disease and timely and regular sharing of information between WHO and its Member States during the pandemic period is essential to determine future severity assessments, if needed.

Future severity assessments would reflect one or a combination of the following factors:

- changes in the virus,
- underlying vulnerabilities, or
- limitations in health system capacities.

The pandemic is early in its evolution and many countries have not yet been substantially affected.

What is WHO doing to respond?

WHO continues to help all countries respond to the situation. The world cannot let down its guard and WHO must help the world remain and become better prepared.

WHO's support to countries takes three main forms: technical guidance, materials support, and training of health care system personnel.

WHO's primary concern is to strengthen and support health systems in countries with less resources. Health systems need to be able to prevent, detect, treat and mitigate cases of illness associated with this virus.

WHO is also working to make stocks of medicines (such as antivirals and antibiotics) and an eventual pandemic vaccine more accessible and affordable to developing countries.

Both antivirals and vaccines have important roles in treatment and prevention respectively. However, existing stocks of antivirals are unlikely to meet the

http://maha-arogya.gov.in

demand. WHO is working closely with manufacturers to expedite the development of a safe and effective vaccine but it will be some months before it is available.

Therefore, rational use of the limited resources will be essential. And medicines are only part of the response. WHO is also deploying diagnostic kits, medicines and masks and gloves for health care settings, teams of scientific experts, and medical technicians so countries in need can respond to local epidemics.

A pandemic sets national authorities in motion to implement preparedness plans, identify cases as efficiently as possible, and minimize serious illness and deaths with proper treatment.

The goal is to reduce the impact of the pandemic on society.

What do I do now? What actions should I look for in my community?

Stay informed. Go to reliable sources of information, including your Ministry of Health, to learn what you can do to protect yourself and stay updated as the pandemic evolves. Community-specific information is available from local or national health authorities.

You can also continue to visit the WHO web site for simple prevention practices and general advice. WHO is not recommending travel restrictions nor does WHO have evidence of risk from eating cooked pork.

SOME IMPORTANT WEBSITES-

www.who.int

www.mohfw.nic.in

www.ndma.gov.in

www.maha-arogya.gov.in

http://maha-arogya.gov.in

A Lesson from History-

The Quarantine order

"Men will be seated not less than three feet apart. Where folding benches are used, only every other seat will be occupied."

"Coughing in all enclosed places will not be tolerated. At Barracks B drill hall, Y.M.C.A. hut, K. of C. hut, and any other places where lectures and entertainments may be held, the Provost marshal will detail sentries to curb the 'coughing habit,' which has become very noticeable at all gatherings."

"It must be remembered that coughing is the means whereby contagious diseases are most effectually spread and men are enjoined to bear this fact in mind whenever they are in enclosed places in company with other men cooperation on the part of every individual at this training station in the simple rule of 'Stop That Coughing' will prevent the inception and spread of disease. "When you want to cough, go out into the open air and do it, and then see the doctor."

New Port Naval Training Station,
Quarantine Order during Spanish Influenza Epidemic-1918.
(Reference- Boston Medical & Surgical Journal, Sept.1918)

http://maha-arogya.gov.in
